# Motor control differs for increasing and decreasing force production during ankle Isometric exercises in children

**DOI:** 10.1186/s13102-023-00727-y

**Published:** 2023-09-23

**Authors:** Qiliang Xiong, Jieyi Mo, Chen Yi, Shaofeng Jiang, Yuan liu

**Affiliations:** 1 Department of Biomedical Engineering, Nanchang Hangkong University, Jiangxi, China; 2 Department of Rehabilitation, Children’s Hospital of Chongqing Medical University, Chongqing, China

**Keywords:** Intramuscular coherence, Force control, Motor development, Children

## Abstract

**Background:**

Performance of the central nervous system (CNS) in increased and decreasing muscle force around the ankle joint is essential for upright tasks of daily living. Previous studies have shown altered CNS control when they decrease force compared with when they increase force in young and older adults. But whether such alteration exists during childhood with incomplete maturation of CNS systems remain unclear. Therefore, this study aimed to evaluate the disparities in intramuscular EMG-EMG coherence, which serve as indicators of corticospinal drive to muscles during ankle isometric increasing and decreasing force generation in children.

**Methods:**

We measured intramuscular EMG-EMG coherence activity of the tibialis anterior (TA) and the associated ability to perform isometric efforts at the ankle in 12 typically developing children (mean ± SD age = 5.91±1.37 years) and 12 healthy young adults (mean ± SD age = 23.16±1.52 years). The participants maintained isometric contractions at 20% of their maximal voluntary contractions (MVC) during ankle dorsiflexion to match a triangle trajectory for 7 s, including ramping up in 3.5 s (increasing force phase) and then linearly ramping down to rest in 3.5 s (decreasing force phase). The variability of force control was characterized by the coefficient of variance (CoV) of force output. Intramuscular EMG-EMG coherence from TA in two frequency bands, the beta band (15–30 Hz) and gamma band (30–45) that could reflect the corticospinal drive, were calculated for the comparison. A repeated measures ANOVA with the within-subjects factor of force generation phase (increasing force vs. decreasing force)x between-subjects factor of the group (children and young adults) was used for statistical analysis.

**Results:**

Regarding the within-subjects difference, our results exhibited significantly higher CoV of force (*p* < 0.01) and lower EMG-EMG coherence of TA when they decrease force compared with when they increase force in both children and young adult groups. Regarding the between-subjects difference, the CoV of force was significantly higher (*p* < 0.01) in children compared to young adults, while the EMG-EMG coherence in children showed a significantly lower (*p* < 0.01) coherence compared with young adults. Furthermore, the EMG-EMG coherence measures were negatively correlated with the CoV of force.

**Conclusions:**

The findings suggest that the age-related development would increase the corticospinal drive to TA muscle to deal with ankle isometric dorsiflexion during childhood, which could be also modulated with the force production phases, including increasing and decreasing force.

## Introduction

Motor performance changes substantially from infancy through childhood as skills are acquired and perfected in a variety of situations and contexts in the completion of activities of daily living [1]. In particular, an important skill acquired during childhood is the ability to modulate increasing and decreasing muscle force based on motor task demands. For example, the ability to increase forces is critical to pick up objects and the ability to decrease forces is essential to release objects [2]. Decreased force steadiness and increased variability of motor performance with aging suggest a different capacity for regulating force production [3]. Furthermore, there is evidence that adults and older exhibit altered force control when they decrease force compared with when they increase force [4]. For instance, participants exhibited altered force control during a power grip task while decreasing force than increasing force, this finding seemed to be independent of the participant’s age [5]. Another study involving isometric contractions of the first dorsal interosseous muscle demonstrated that older adults exhibited greater force fluctuations during the release force phase of the task [6]. Taken together, it is well accepted that aging impairs motor control for increasing and decreasing muscle force and leads to different motor performance, including increased force variability, However, whether such disparities exist during childhood with incomplete maturation of CNS systems remains largely unknown.

Differences in motor performance during childhood may be associated with age-related changes in muscle properties, as developmental changes have been identified in mean power frequency [7], M-wave features [8], and metabolism [9] of muscles during task-dependent muscle regulation. Significant changes in the central nervous system (CNS) throughout development also play a potential role in the age-related differences in force modulation. Whereas most corticospinal terminations in adults are contralateral to their origin in the cortex, during development they also terminate extensively in the ipsilateral gray matter and branch more at the spinal cord, which may cause less effective motor unit (MU) recruitment via CNS. For instance, activation of spinal motor circuits becomes more synchronous due to myelination [10] and maturation of spinal synapses, as demonstrated by decreased transcranial magnetic stimulation motor threshold throughout early development [11, 12]. Despite these insights into the CNS’s maturation during development, there remains limited information on CNS’s motor control mechanisms that contribute to differences in motor performance between increasing and decreasing muscle force in typical developing children.

To fill this gap, this study aimed to evaluate the CNS mechanisms underlying muscle activation and their role in overall motor performance in children. Specifically, we chose to investigate the increasing and releasing force control of dorsiflexion (tibialis anterior, TA), which is essential for gait [13] and balance [14]. Understanding the underlying contribution of the nervous system development to control dorsiflexion is of great significance. Furthermore, ankle dorsiflexion is more controlled by the sensorimotor cortex associated with fine motor movements [15]. The motor control command that produces an increasing and releasing force is sent from the motor cortex to the muscle through the corticospinal tract [16]. The functional integrity of this corticospinal linkage can be detected in coherence between EMG signals in synergistic muscles or within the same muscle [17, 18]. This method allows for more direct observation of the output of the CNS, while previous research has suggested that such inter- and intramuscular coherence in beta (15–30 Hz) and gamma (30–45 Hz) bands are predominantly driven by the motor cortex [17]. Further, the findings of beta-band and gamma-band coherence increases from childhood to middle age have been consistently demonstrated in studies examining corticomuscular and intermuscular coherences [17, 19]. However, these studies mainly utilized a steady force production approach, and little information exists about the effects of age on increasing and decreasing force control during childhood. Notably, this study offers a novel viewpoint, focusing specifically on the disparities in increasing and decreasing force control during childhood. These differences can be effectively quantified with EMG-EMG coherence, offering invaluable insights into the CNS mechanisms underpinning ankle dorsiflexion in typical developing children.

Therefore, in the current study, we measured the beta band and gamma band of intramuscular coherence of tibialis anterior (TA) and associated force variability to perform isometric increasing and decreasing efforts at the ankle in children and young adults. We hypothesize that children will exhibit lower force control ability of the ankle dorsiflexion when compared to young adults. It was additionally expected that the force variability would be significantly increased during the decreasing phase of contraction in comparison to the increasing phase of contraction.

## Materials and methods

### Participants

Twelve children participants (ages 4–8 years, 5.91 ± 1.37 years) and 12 young adults (ages 21–25 years, 23.16 ± 1.52 years) volunteered for the study. The sample size calculations were derived from power analysis and sample size software (PASS, NCSS Statistical Software, Kaysville, UT, USA), where the alpha and minimum required power is fixed at 0.05 and higher than 0.90 respectively. The values of pooled standard deviation and difference of means of the two groups used during the estimation calculations were obtained from a prior study. All participants had no reported neurological disorders or previous history of lower limb pathology or surgery. We first determined the dominant leg using a battery of clinical assessments, including the unipedal stance test [20], their preferences for kicking a ball, and protective step in response to a perturbation. The summarized demographic information of the participants is listed in Table 1. The study was approved by the ethics committee of the children’s hospital of Chongqing medical university (065/2011). All participants completed informed consent, or parental consent, and participant assent for participants younger than 18 years, before participation in the protocol.

**Table 1:** Participant demographic information

	Children	Young adults
Participants	12(6 M, 6 F)	12 (7 M, 5 F)
Age(years)	5.91±1.37	23.16±1.52
Body Height(cm)	119.5±9.1	169.7±8.2
Body mass (Kg)	23.2±4.4	62.9±10.2
Leg length(cm)	26.1±1.6	41.0±3.2
MVC(N)	11.0±2.1	41.5±12.7

*MVC *Maximum voluntary contraction (MVC) during dorsiflexion

### Experimental procedure

#### Experimental setup

**Fig. 1:**
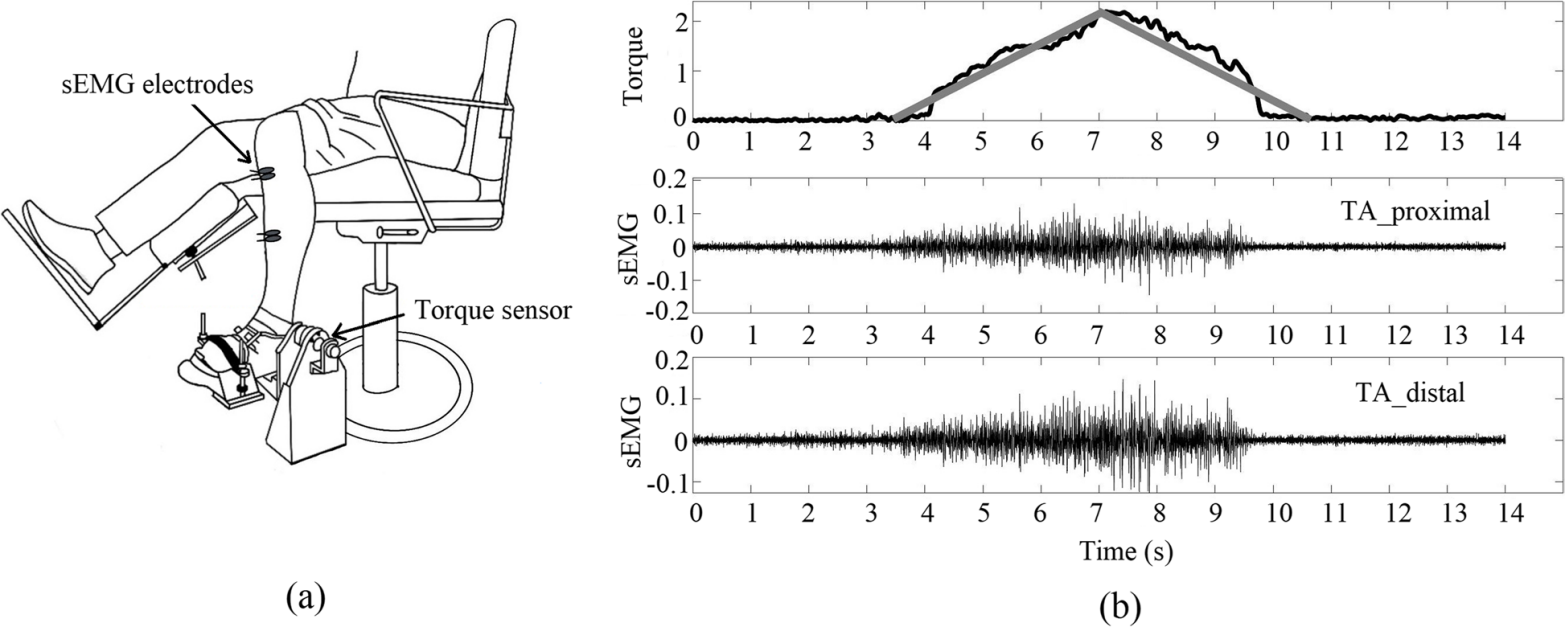
**a** A representative example of one participant’s foot secured to the footplate with the sEMG electrodes placed over the tibialis anterior (TA) muscles. **b** A representative example of the participant’s force output and the corresponding EMG signals collected from the proximal and distal end of the TA muscle. The grey line is the target line as 20% of maximal voluntary contraction (%MVC) that participants need to follow, including ramping up in 3.5 s and then linearly ramping down to rest in 3.5 s. The black line is the participant’s force output and the corresponding EMG signal

The participants were seated comfortably with their trunk reclined 15º from vertical and supported by shoulder straps, which resulted in participant position of 90º hip flexion and neutral frontal plane alignment. This position was both comfortable and stable for the participant. The foot of the participant’s dominant leg was rigidly attached to a customized footplate, secured by a custom set of padded rigid bars and straps (see Fig. 1). The foot plate’s position was adjusted to align the rotation axis of the torque sensor (HCNJ-101, Haibohua, China), with the knee at 90º of extension and the ankle was at neutral.

A surface EMG system (ME6000, Mega Electronics Ltd, Finland) was used to measure the surface EMG activity from tibialis anterior (TA) muscles. The skin was prepared (shaving, cleaning with an abrasive cream to remove dead skin cells, then washing with warm water). After skin preparation, recording electrodes were placed at the proximal and distal end of the TA muscle respectively. Reference electrodes were positioned at the ankle and tibial tuberosity. EMG signals were sampled at 2000 Hz, converted to digital data, and bandpass filtered at 10–500 Hz.

#### Experimental protocol

After a warm-up procedure, participants were first instructed to produce a maximal voluntary effort (maximum voluntary contraction, MVC) in dorsiflexion, and maintain the maximal effort for 3 s. Compensatory movements were visually monitored. In the event of compensation, the participant was coached, and the trial was repeated. At least three MVC trials were completed for the dorsiflexion task with a between-trial break of 2 min. When the highest MVC was the last trial, additional trial(s) were taken to account for learning. This phenomenon was rare in the adult group, but three young children were tested more than three times in the MVC test. The highest MVC value was used as the normalization reference for the submaximal force levels. After a brief rest (~ 3 min), participants were asked to generate isometric submaximal ramp torque profiles. A successful trial included identical force profiles of a linear ramp for 3.5 s and decreasing linearly for 3.5 s in triangle form (see Fig. 1). Dorsiflexion efforts are guided by visual feedback showing the generated and targeted force. Participants were given 1–4 trials to familiarize themselves with the required task. After training, participants completed a total of 8 trials.

### Data analysis

#### Force variability

**Fig. 2:**
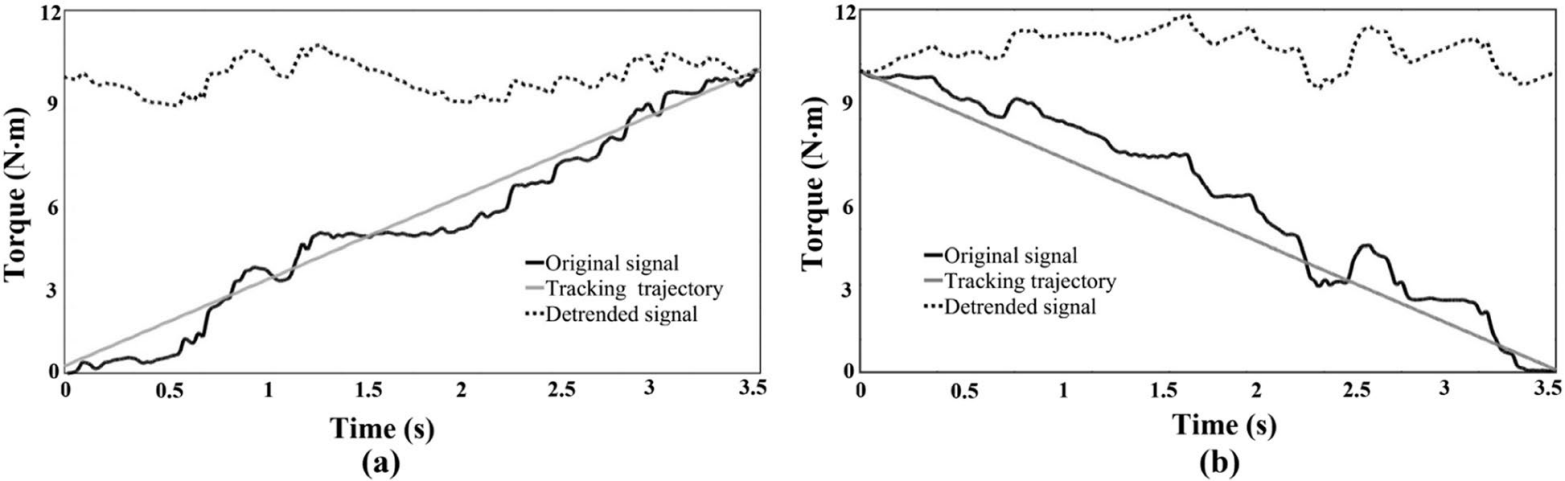
A representative trial showing detrended force outputs during increasing force (**a**) and decreasing force (**b**) phases

Data were processed and analyzed offline using MATLAB (MathWorks Inc, USA). The force signal was low-pass filtered offline at 20 Hz. The coefficient of variance (CoV) of the detrended force signal was then used to quantify force variability (as shown in Fig. 2). We were able to erase the linear trend and drifting of force from the data by detrending the force signal, which might have influenced the estimation of force variability.

#### EMG-EMG coherence

To improve the reliability of the coherence estimations, the electromyography (EMG) signals from 8 trials for each participant were first concatenated into a single continuous time series. The coherence analysis was carried out using the procedures described by Halliday [21]. The raw data was visually reviewed, and any signals that had disruption or high noise were excluded from further analysis. Then, the surface EMG signals were full-wave rectified and the coherence function was derived using the following equation for the two signals x and y (i.e., EMG signals).

$${\left|{C}_{xy}\left(f\right)\right|}^{2}=\frac{\left|{P}_{xy}\left(f\right)\right|}{{P}_{xx}\left(f\right){P}_{yy}\left(f\right)}$$

where ${P}_{xy}\left(f\right)$is the cross-spectra and ${P}_{xx}\left(f\right)$ and ${P}_{yy}\left(f\right)$ are the auto-spectra of the signals x and y, respectively. The coherence was calculated for a segment length of 2000 samples with 50% overlap, using a Hanning window. To determine the statistical significance of coherence estimates, we used the significance limit of the null distribution, which was calculated according to the method proposed by Rosenberg et al. [22]. The formula used to calculate the significance limit for zero coherence at α = 0.05 was based on the number of disjoint segments (L):

$$Sig\left(\alpha \right)=1-{(1-\alpha )}^{\frac{1}{L-1}}$$

To compare coherence estimates between participants, we applied the arc hyperbolic tangent transformation (Fisher transformation) to all coherence estimates, following the method proposed by Rosenberg et al. [22].

$${\text{tanh}}^{-1}\left(x\right)=0.5\cdot \text{log}\frac{(1+x)}{(1-x)}$$

**Fig. 3:**
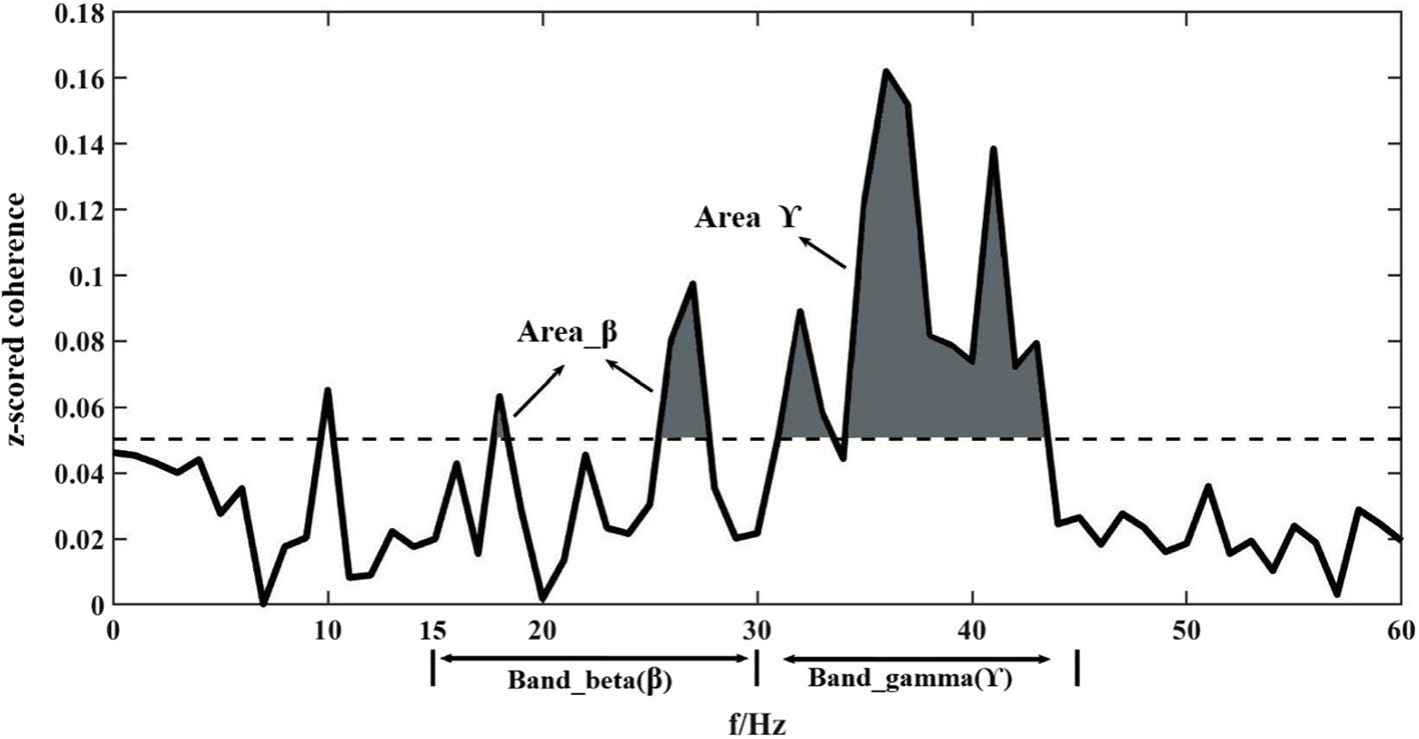
A representative example showing the area below the z-scored coherence profile within the frequency band of beta (Area_β) and gamma (Area_ϒ)

To analyze the frequency distribution of correlated neural inputs, we identified frequency intervals exhibiting significant z-scored coherence estimates among participants. In Fig. 3, a typical z-scored coherence profile and significance level obtained from a pair of EMG signals are presented as an example. We first calculated significant z-scored coherence estimates in the beta-band (15–30 Hz) and gamma-band (30–45 Hz) frequency ranges. then, we computed the integrals for z-scored coherence estimate profiles over a frequency band that included all coherence estimates exceeding the significance level of the null distribution. The strength of corticospinal command was represented by the area below the z-scored coherence profile within the beta (Area_β) and gamma (Area_ϒ) frequency bands, respectively.

### Statistical analysis

A repeated measures ANOVA with the within-subjects factor of force production phases (increasing force vs. decreasing force)x between-subjects factor of group (children and young adults), was used for the comparisons of EMG-EMG coherence and CoV of force. A Bonferroni corrected post-hoc test was used if there were significant main effects. To evaluate the relationship between the CoV of force as a dependent variable and the Area_β and Area_ϒ as independent variables, Spearman’s correlation analysis was completed. For the correlation analysis, the strength of correlation was interpreted as weak (*R* = 0-0.2), mild (*R* = 0.2–0.4), moderate (*R* = 0.4–0.6), strong (*R* = 0.6–0.8), or very strong (*R* = 0.8–1.0). All analyses were performed using the SPSS (v18.0, SPSS Inc, USA). For all statistical tests, results were considered statistically significant when *p* < 0.05.

## Results

### Force variability

**Fig. 4:**
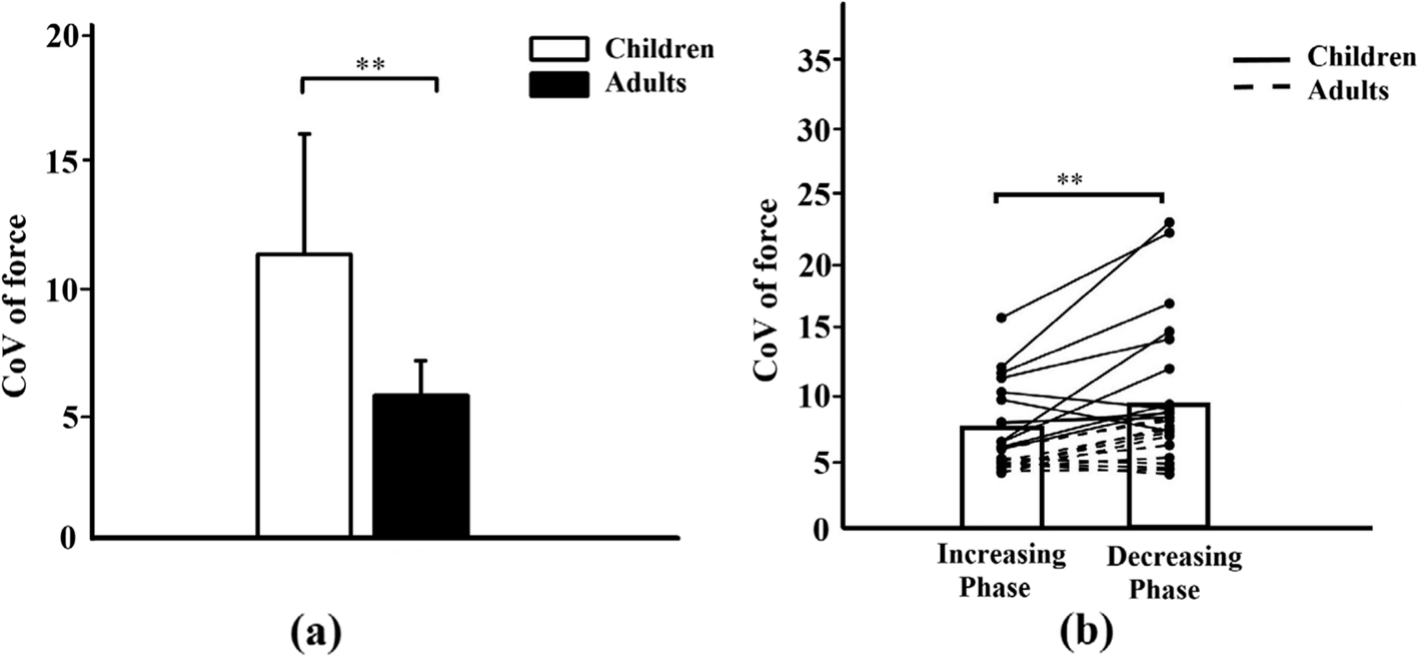
**a** Children exhibited greater CoV of force compared with young adults; **b** Dorsiflexion during force decreasing phase exhibited greater CoV of force compared with the force increasing phase. **p < 0.01

There was a significant effect of group (F = 21.872, *p* < 0.001, partial eta squared = 0.499) on the CoV of force, with children exhibiting significantly greater CoV than young adults (*p* < 0.001), as shown in Fig. 4a. The effect of force production phase on the CoV of force was also significant (F = 16.547, *p* < 0.01, partial eta squared = 0.429). The post-hoc analysis indicated that the CoV of force during decreasing force phase was significantly higher (*p* < 0.01) than increasing force phase, as shown in Fig. 4b. No significant interactions between the group and force production phase on the CoV of force (F = 3.497, *p* = 0.075, partial eta squared = 0.137) was detected.

### EMG-EMG coherence

#### Area_β

**Fig. 5:**
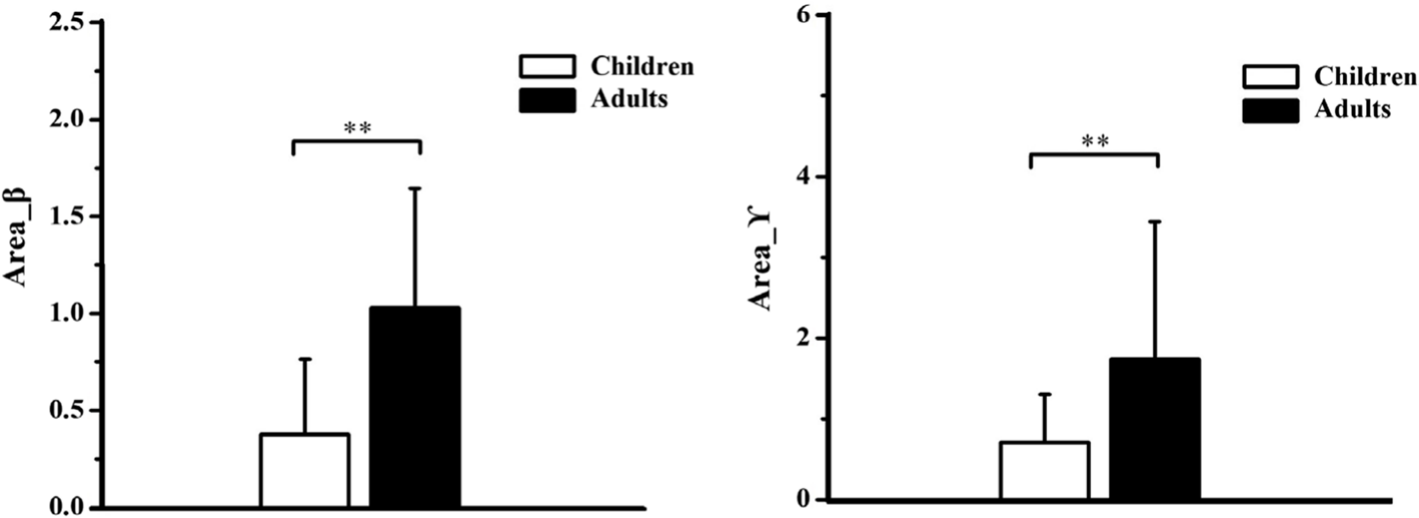
Children exhibited significantly lower Area_β and Area_ϒ than young adults. *p < 0.05, **p < 0.01

**Fig. 6:**
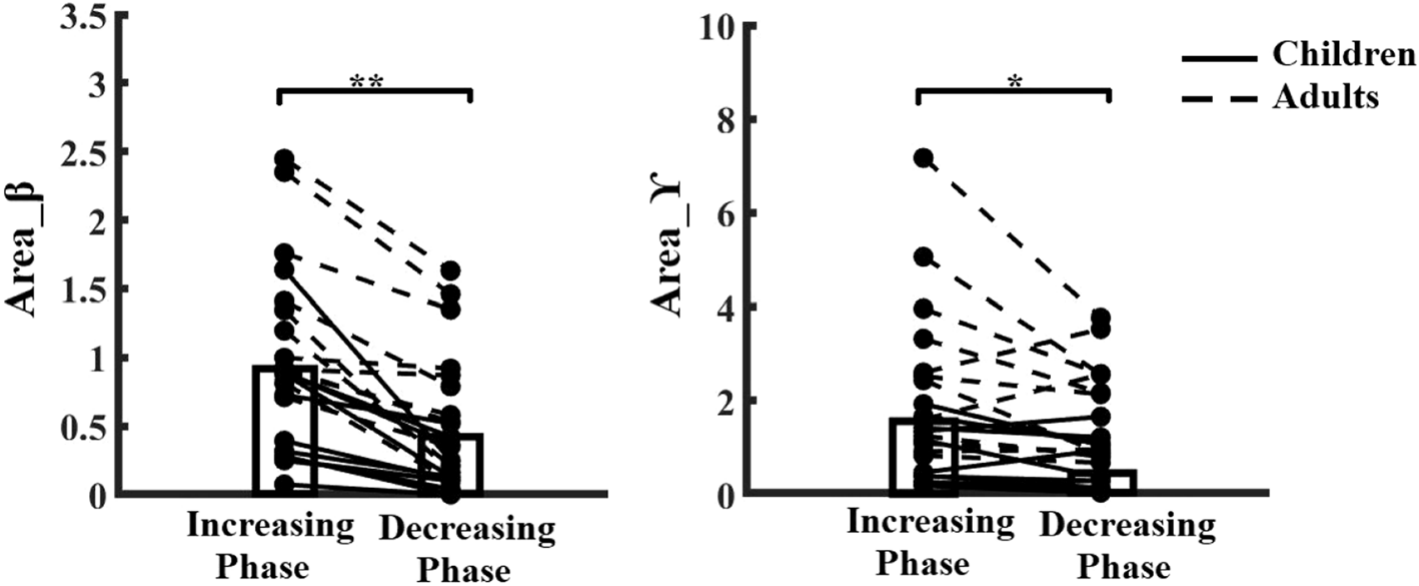
Area_β and Area_ϒ during decreasing force phase were both significantly lower than increasing force phase. *p < 0.05, **p < 0.01

There was a significant effect of group (F = 14.367, *p* < 0.01, partial eta squared = 0.395) and force production phase (F = 41.478, *p* < 0.001, partial eta squared = 0.653) on the Area_β. The post-hoc test found that children exhibited significantly lower Area_β than young adults (*p* < 0.01), as shown in Fig. 5, while Area_β during increasing force phase was significantly higher than decreasing force phase (*p* < 0.001), as shown in Fig. 6. No other significant main effect or interaction (F = 1.403, *p* = 0.249, partial eta squared = 0.06) was reported.

#### Area_ϒ

Similarly, there was a significant effect of group (F = 13.841, *p* < 0.01, partial eta squared = 0.386) and force production phase (F = 5.977, *p* < 0.05, partial eta squared = 0.214) on the Area_ϒ. The post-hoc test found that children exhibited significantly lower Area_ϒ than young adults (*p* < 0.01), as shown in Fig. 5, while Area_β during increasing force phase was significantly higher than decreasing force phase (*p* < 0.05), as shown in Fig. 6. No other significant main effect or interaction (F = 2.214, *p* = 0.159, partial eta squared = 0.088) was reported.

### Associations between force variability and intramuscular coherence

**Fig. 7:**
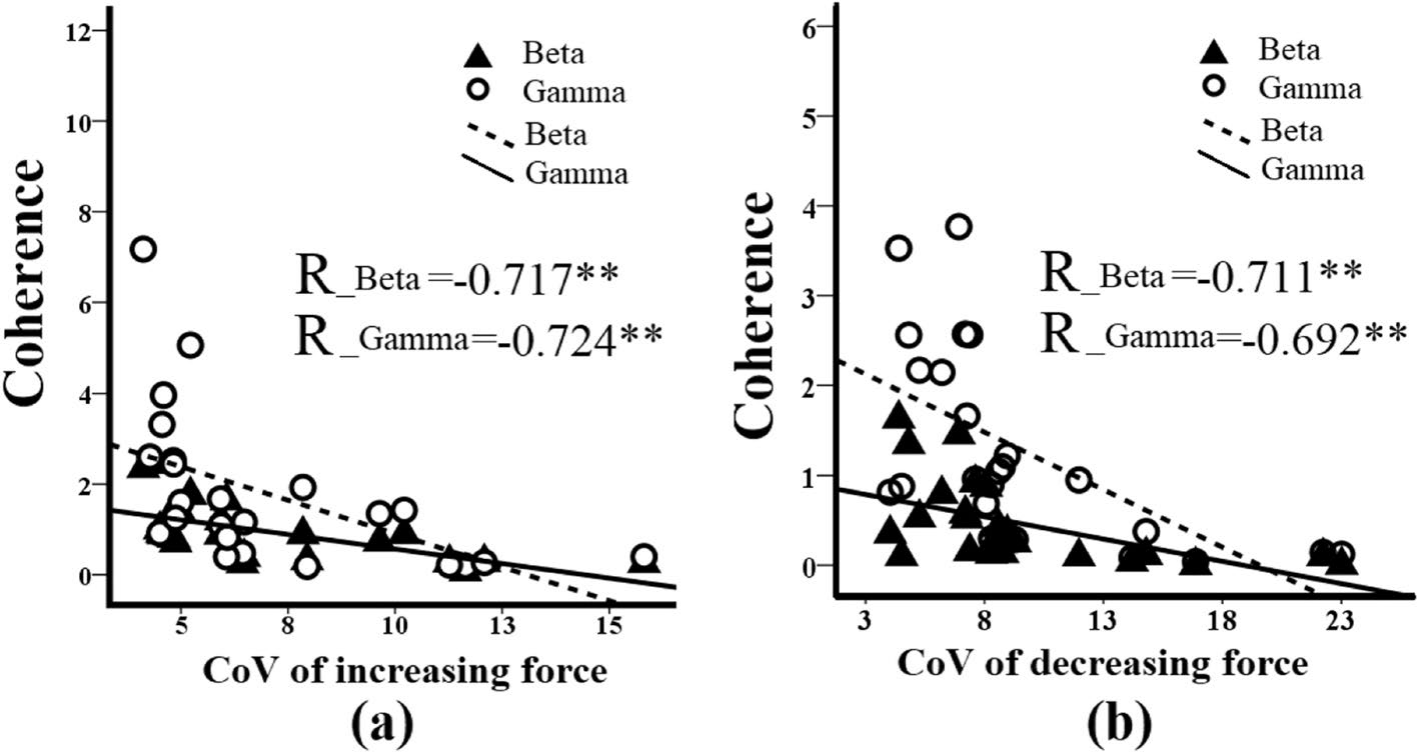
Associations between force variabilities and intramuscular EMG-EMG coherence measures. Specifically, subplots (a) show the associations between the CoV during force increasing phase and Area_β and Area_ϒ, respectively. Subplot (b) shows the associations between the CoV during the force decreasing phase and Area_β and Area_ϒ, respectively. ** *p* < 0.01

With regards to the decreasing force phase (as shown in Fig. 7a), the CoV of force was strongly associated with TA intramuscular coherence (R=-0.717 for Area_β; R=-0.724 for Area_ϒ). With regards to the force decreasing phase (as shown in Fig. 7b), the CoV of force was strongly associated with TA intramuscular coherence (R=-0.711 for Area_β; R=-0.692 for Area_ϒ). Detailed regression results are shown in Fig. 7.

## Discussion

To our knowledge, this is the first study to document the difference in controlling increasing and decreasing force generation in children’s ankle dorsiflexion tasks. In addition to the greater force variability and lower intramuscular coherence in children compared with young adults, which has been recently reported, our results demonstrated a higher force variability and decreased coherence during decreasing force phase relative to increasing force phase. Furthermore, both the beta band and gamma band of intramuscular coherence were negatively correlated with the force variability. These results offered additional insight into the CNS’s mechanisms leading to increasing and decreasing force generation during childhood development.

### Effect of age

Our results generally support the finding that young adults had a better ability to control “force” than children (Fig. 4a). This finding is in line with previous developmental studies on force steady control in typically developing children, where younger children are typically more variable than adults in controlling a stable force [23]. Such differences in the ability of force control between children and adults cannot be entirely explained by the growth of muscle strength and size, but are also related to the maturation of the CNS during development, and have been suggested to follow a time course of corticospinal maturation [24]. This developmental change can be reflected in the CNS output, as measured by the beta band of intramuscular EMG coherence in this study.

Another major finding in this study was that lower intramuscular coherence was recorded in children compared with young adults(as shown in Fig. 5), which is in line with findings during steady isometric tasks and gait [17, 19]. The increased coherence observed during development from childhood to adulthood can be attributed to various neural mechanisms. For example, James et al. demonstrated that EEG-EMG coherence in the beta range between the motor cortex and forearm extensor muscles increases with age [25], indicating age-dependent changes in the synchronous corticospinal drive. Our results support and expand upon this previous work by suggesting that this descending cortical involvement in the control of ankle muscle activity progressively increases as a function of childhood development.

Combined with the force and coherence results, we confirmed that children exhibited greater variability in force control when generating increasing and decreasing forces, reflecting the developmental status of the CNS. The reduced intramuscular coherence may be related to a less organized neural drive [26] compared to that of typical adults. The cortex is still undergoing maturation during the age range examined [17], and as the CNS matures from childhood to early adulthood through the process of synapse strengthening and elimination [27, 28], strength in neural drive to the muscle is enhanced [29].

### Effect of force production phase

Moreover, we conducted an exploratory analysis to investigate the effects of the force production phase on force variability and intramuscular coherence. Our results revealed that children exhibited greater force variability and decreased coherence during decreasing force phase as compared to increasing force phase (as shown in Fig. 6). The differences during force increasing and decreasing could be attributed to the neurophysiological mechanisms underlying the generation and release of forces. Further, we conducted an exploratory analysis to investigate the effects of the force production phase on force variability and intramuscular coherence. Our results revealed that children exhibited greater force variability and lower coherence during decreasing force phase as compared to increasing force phase. Moreover, Park et al., (2016) demonstrated that increased force variability during decreasing force phase is caused by reduced modulation of multi-motor unit discharge rate in the 35 to 60 Hz frequency range [30]. Thus, altered activation and modulation of motor units during force decrement compared with increment influences force control. In the present study, the beta (15–30 Hz) and gamma (30–45 Hz) of TA intramuscular coherence reflects common oscillatory drive to motor neuron pools from the sensorimotor cortex, resulting in the modulation of motor units. That is, the difference in intramuscular coherence between increasing and decreasing force phases during development may provide a window into the different courses of developmental processes associated with the maturation of descending common oscillatory drive to motor neurons.

Furthermore, a recent investigation into brain activation patterns during controlled force production observed that the process of force reduction is not simply characterized by the deactivation of specific brain regions involved in force generation, but rather is governed by the systematic activation of alternate brain regions [31]. Specifically, cortical and subcortical regions including the primary motor cortex, supplementary motor area, and basal ganglia are known to be involved in force production. In contrast, force reduction was facilitated by heightened activation and additional recruitment of alternative brain regions such as the ipsilateral dorsolateral prefrontal cortex, and reduced activity in the bilateral anterior cingulate cortex [31]. These findings indicate that distinct neural substrates govern the execution of controlled force generation and reduction.

### Associations between intramuscular coherence and force variability

According to previous research, variability in force generation may be linked to differences in the common drive to the motor neuron pool [32]. Looking at our results, force variability during dorsiflexion was associated with the beta band and gamma band of TA intramuscular coherence (as shown in Fig. 7), suggesting that participants with higher intramuscular coherence also exhibited greater force control precision. Such findings are similar to those previously reported in children’s walking tasks. For instance, Petersen et al. found a significant age-related decrease in step-to-step variability of the position during the swing phase of walking, which was correlated with increased gamma band coherence during walking [17]. Our results suggest that during childhood development, there is an age-related increase in oscillatory corticospinal input to the ankle muscle motoneuron pools, potentially impacting the maturation of precision force control. From the perspective of predictive coding theory [33], we suggest that our findings may reflect an age-related increase in reliance on feedforward control as the developing nervous system becomes more adept at predicting the sensory consequences of movement. Our results could inform the development of new intervention strategies aimed at improving sensorimotor control in children with central motor disorders.

### Limitations and future directions

We acknowledge several limitations of our study. Firstly, the potential cross-talk between pairs of EMG recordings is a specific concern, primarily due to the small muscle size and the corresponding small distances between the pair of electrodes in young children. Cross-talk from closely positioned electrodes would produce the opposite result. To mitigate this, we used tiny bipolar electrodes with limited recording areas, placing them as far apart as anatomically possible. We also excluded data with narrow peaks in frequency-coherence plots or consistent, significant coherence across all frequencies (0-400 Hz), typically suggestive of cross-talk influence [34]. Overall, we are confident that potential cross-talk did not substantially alter our results. Secondly, the limited sample size for the correlation analysis is another limitation in the current study, and more subjects are required to enhance the significant correlations in future studies. Finally, we were unable to directly measure corticospinal contributions, which precludes us from ruling out the influence of sensory afferent input and other neural drives via descending pathways such as subcortical levels on intramuscular coherence changes. Nevertheless, the representativeness of the identified intramuscular coherence is expected to be equal between different groups or different tasks due to the consistency of assumptions about the algorithm in both children and adults participants. Therefore, the comparisons of intramuscular coherence are still worthwhile. The overall balance between these assumptions and participant comfort is still weighted towards embracing these techniques to understand typical development better and provide a key comparison to children with central nervous system injuries.

Given that performance of the CNS in force increasing and decreasing in muscles around the ankle joint is essential for upright tasks of daily living. Moreover, previous studies have demonstrated that modifications in beta and gamma frequency bands of EMG-EMG coherence are linked with corticospinal tract damage during CP’s gait and static contraction tasks [35–37]. A greater understanding of these features in children with typical development can also be understood in the context of those with early central and peripheral injuries. Indeed, our results suggest differential control of isometric force control during decreasing force phase versus increasing force phase in children. Future work should examine age and disease-related changes in isometric force control. For example, neurological conditions such as cerebral palsy, may provide critical insights into control of forces [38, 39]. A direct relationship between motor cortex activities (EMG-EMG coherence) and isometric task performance found in the current study, suggests that improving the ability of isometric increasing and decreasing force control could be a potential strategy for motor rehabilitation to facilitate motor performance. That is, according to the neuronal plasticity of the motor system, training the ankle’s dorsiflexion to modulate force increasing and decreasing may promote CNS recovery in children with neurological disease.

## Conclusion

In conclusion, the findings suggest that compared to young adults, incomplete maturation of the neuromuscular system in children results in lower intramuscular EMG-EMG coherence of TA muscle, which may further contribute to higher force variability during ankle isometric increasing and decreasing force generation. Notably, this study also suggests that CNS control pathways of increasing and decreasing force production may have different courses of development during childhood. These findings shed light on the neural mechanisms underlying increasing and decreasing force control and motor development in children and may inform the development of targeted interventions for children with neurological motor disorders.

## Data Availability

The datasets generated and/or analyzed during the current study are not publicly available due clinical policy but are available from the corresponding author on reasonable request.
